# Brefeldin A and M-COPA block the export of RTKs from the endoplasmic reticulum *via* simultaneous inactivation of ARF1, ARF4, and ARF5

**DOI:** 10.1016/j.jbc.2024.107327

**Published:** 2024-04-26

**Authors:** Miyuki Natsume, Mariko Niwa, Sho Ichikawa, Takuma Okamoto, Hisazumi Tsutsui, Daiki Usukura, Takatsugu Murata, Ryo Abe, Motoyuki Shimonaka, Toshirou Nishida, Isamu Shiina, Yuuki Obata

**Affiliations:** 1 Laboratory of Intracellular Traffic & Oncology, National Cancer Center Research Institute, Chuo-ku, Tokyo, Japan; 2 Faculty of Science, Department of Applied Chemistry, Tokyo University of Science, Shinjuku-ku, Tokyo, Japan; 3 Faculty of Science, Department of Chemistry, Tokyo University of Science, Shinjuku-ku, Tokyo, Japan; 4 Tokyo University of Science, Noda, Chiba, Japan; 5 National Cancer Center Hospital, Chuo-ku, Tokyo, Japan; 6 Laboratory of Nuclear Transport Dynamics, National Institutes of Biomedical Innovation, Health and Nutrition, Ibaraki, Osaka, Japan

**Keywords:** RTK, KIT, EGFR, MET, GEF, ARF, Golgi/TGN, endoplasmic reticulum, BFA, M-COPA

## Abstract

Normal receptor tyrosine kinases (RTKs) need to reach the plasma membrane (PM) for ligand-induced activation, whereas its cancer-causing mutants can be activated before reaching the PM in organelles, such as the Golgi/*trans*-Golgi network (TGN). Inhibitors of protein export from the endoplasmic reticulum (ER), such as brefeldin A (BFA) and 2-methylcoprophilinamide (M-COPA), can suppress the activation of mutant RTKs in cancer cells, suggesting that RTK mutants cannot initiate signaling in the ER. BFA and M-COPA block the function of ADP-ribosylation factors (ARFs) that play a crucial role in ER–Golgi protein trafficking. However, among ARF family proteins, the specific ARFs inhibited by BFA or M-COPA, that is, the ARFs involved in RTKs transport from the ER, remain unclear. In this study, we showed that M-COPA blocked the export of not only KIT but also PDGFRA/EGFR/MET RTKs from the ER. ER-retained RTKs could not fully transduce anti-apoptotic signals, thereby leading to cancer cell apoptosis. Moreover, a single knockdown of ARF1, ARF3, ARF4, ARF5, or ARF6 could not block ER export of RTKs, indicating that BFA/M-COPA treatment cannot be mimicked by the knockdown of only one ARF member. Interestingly, simultaneous transfection of ARF1, ARF4, and ARF5 siRNAs mirrored the effect of BFA/M-COPA treatment. Consistent with these results, *in vitro* pulldown assays showed that BFA/M-COPA blocked the function of ARF1, ARF4, and ARF5. Taken together, these results suggest that BFA/M-COPA targets at least ARF1, ARF4, and ARF5; in other words, RTKs require the simultaneous activation of ARF1, ARF4, and ARF5 for their ER export.

Ligand-bound receptor tyrosine kinases (RTKs) on the plasma membrane (PM) autophosphorylate tyrosine residues specifically and recruit docking proteins to these phospho-sites to activate downstream signaling cascades, such as the RAS-extracellular signal-regulated kinase (RAS-ERK) pathway, the phosphatidylinositol-3 kinase-AKT (PI3K-AKT) pathway, and signal transducer and activator of transcription proteins (STATs), resulting in cell proliferation, survival, and differentiation (1, 2). Therefore, constitutively active mutants of RTKs, such as epidermal growth factor receptor^ΔEX19^ (EGFR^ΔEX19^), KIT^ΔEX11^, and FMS-like tyrosine kinase three internal tandem duplication (FLT3-ITD), can cause autonomous proliferation of host cells (3, 4, 5). EGFR, KIT, and FLT3 mutants are well known as major oncogenic drivers of lung adenocarcinoma (LAD), gastrointestinal stromal tumor (GIST)/mast cell leukemia (MCL), and acute myeloid leukemia (AML), respectively (3, 4, 5, 6, 7). RTKs are type I transmembrane proteins whose carboxy-terminal tyrosine kinase domain is oriented towards the cytosolic side (1, 2). Soon after their synthesis in the ER, RTKs undergo partial glycosylation and subsequently move to the Golgi for complex glycosylation, followed by trafficking toward the PM (8).

Recently, we found that, unlike normal RTKs, constitutively active RTK mutants, such as KIT and FLT3, are aberrantly retained in the Golgi/*trans*-Golgi network (TGN) and endosome-lysosome compartments, which serve as their oncogenic signaling platforms, leading to autonomous cell growth (9, 10, 11, 12, 13). In addition, RTKs in the endoplasmic reticulum (ER) are unable to transduce signals (12, 13, 14, 15), suggesting that blockade of ER export is a new strategy for inhibiting the growth signaling induced by RTK mutants.

ADP-ribosylation factors (ARFs) are small GTPase proteins that serve as a molecular switch of intracellular protein trafficking, such as ER export and endocytosis (16, 17). ARFs are GTP-bound and active on membranes and GDP-bound and inactive in the cytosol (17). Guanine nucleotide exchange factors (GEFs) activate ARFs by substituting GDP in ARFs with cytosolic GTP (16). Human ARFs and GEFs are composed of 29 and 15 members, respectively (16, 17). Brefeldin A (BFA) is a well-known inhibitor that blocks protein transport from the ER to the Golgi by immobilizing GEF-ARF complexes (18, 19, 20). In our previous studies, we demonstrated that KIT and FLT3 cannot move to the Golgi from the ER in the presence of BFA (9, 10, 13), indicating that RTKs move from the ER in a GEF-ARF-dependent manner. However, the specific GEF-ARF members that play a critical role in the biosynthetic transport of RTKs from the ER remain uncertain.

Although the potential of BFA as an anti-cancer drug has been investigated (21), its stability is considerably low *in vivo* and its development as a clinical drug has not progressed. On the other hand, 2-methylcoprophilinamide (M-COPA), which similarly blocks the GEF-ARF complex (22, 23), has higher bioavailability and thus is more efficient *in vivo* (24, 25). We recently confirmed using *in vitro* experiments that M-COPA suppresses the growth signaling of KIT/FLT3 mutants in GIST and AML cells by blocking the ER export of these mutants (12, 13, 14, 15). Therefore, the development of M-COPA and its derivatives as anti-cancer drugs has been initiated currently. Although the mechanism of action is important for establishing these compounds as anti-cancer drugs, which GEF/ARF members are negatively affected by M-COPA is still uncertain. Moreover, inhibition of ER–Golgi transport induces ER stress signaling, which is involved in apoptosis (26). It remains unknown whether ER stress signaling is essential for cell growth suppression/apoptosis in cancer cells expressing an RTK mutant, such as KIT in GISTs and EGFR in LAD.

The present study aimed to determine the GEF-ARF members that play a key role in the export of RTKs from the ER by investigating the mechanism of action of M-COPA in inhibiting the growth of RTK-addicted cancer cells. Further, we attempted to identify the GEF and ARF proteins that are affected by M-COPA.

## Results

### M-COPA inhibits the trafficking of RTKs from the ER

**Figure 1:**
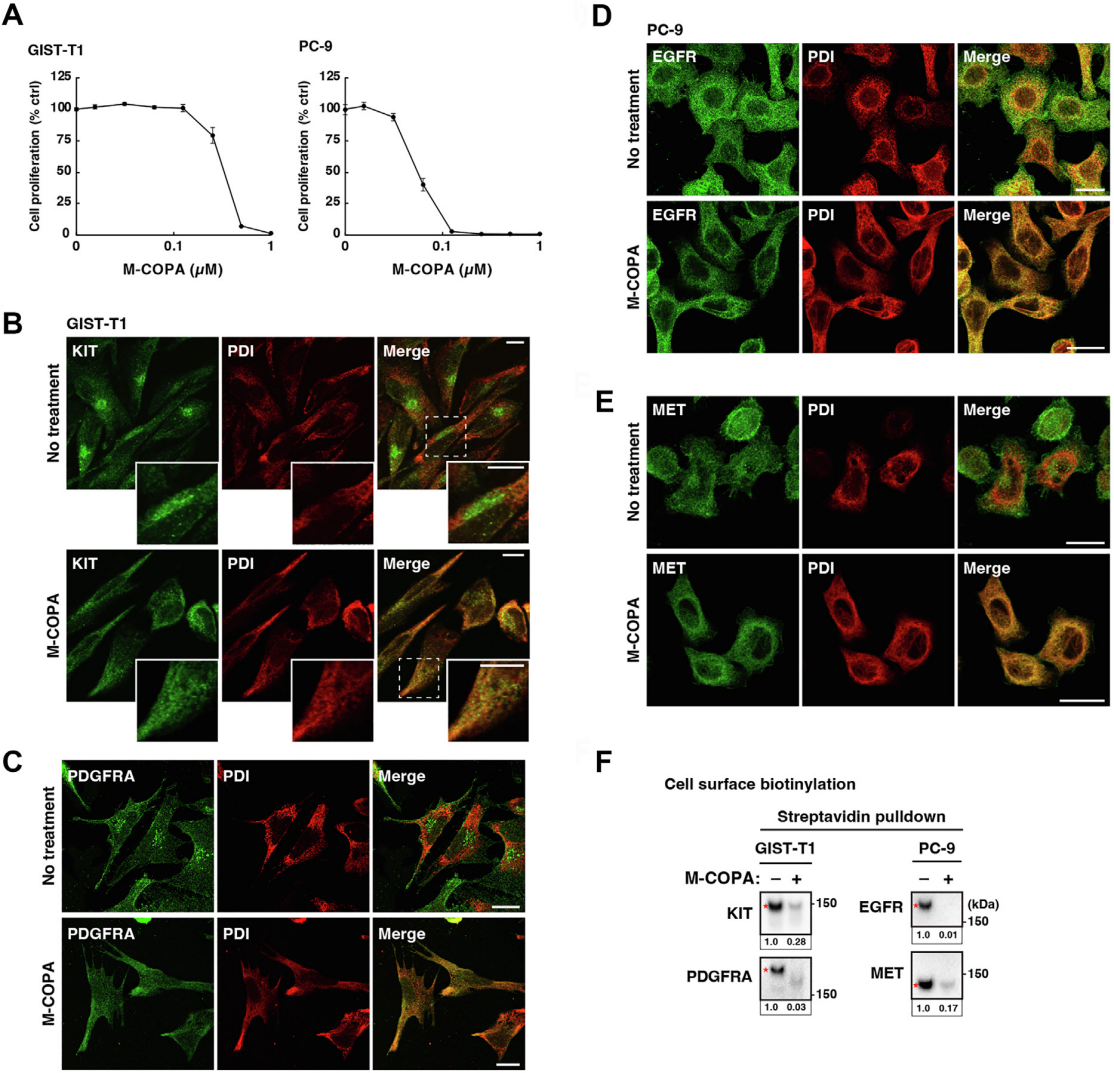
**M-COPA blocks ER export of RTKs, such as KIT, PDGFRA, EGFR, and MET.***A*, GIST-T1 and PC-9 cells were treated with M-COPA for 48 h. Cell proliferation was assessed by ATP production. Values represent mean ± SD (*n* = 3). *B*–*F*, GIST-T1 (*B* and *C*) and PC-9 cells (*D* and *E*) were treated for 8 h with M-COPA at concentrations of 1 and 0.2 μM, respectively. *B*–*E*, cells were immunostained for protein disulfide isomerase (PDI, ER marker) in conjunction with KIT, PDGFRA, EGFR, or MET. Magnified images of the boxed area are shown. Scale bars indicate 10 μm and 20 μm in (*B*) and (*C*–*E*), respectively. *F*, cell surface proteins were treated with biotin. The biotinylated proteins were pulled down with streptavidin and subsequently immunoblotted for KIT, PDGFRA, EGFR, and MET. *Red asterisks* indicate the cell surface RTKs. Relative band intensities normalized with each control sample are shown. Molecular size markers in kDa are indicated on the *right*.

To examine whether ER export of RTKs other than KIT was blocked by M-COPA, we used GIST-T1 cells (a human GIST cell line expressing mutant KIT (KIT^Δ560–578^) and wild-type platelet-derived growth factor receptor A (PDGFRA)) (27) and PC-9 cells (a human LAD cell line expressing mutant EGFR (EGFR^Δ746–750^) and wild-type hepatocyte growth factor receptor (MET)) (28). Cell proliferation assay, which measures ATP levels as a growth index (29), showed that M-COPA completely suppressed the proliferation of GIST-T1 and PC-9 cells at 1 μM and 200 nM concentrations, respectively (Fig. 1*A*). Immunofluorescence confocal microscopic analysis revealed that under control conditions, KIT and PDGFRA in GIST-T1 cells were found at the perinuclear region together with Golgi markers (Figs. 1*B* and S1*A*) (10, 11, 15); however, these RTKs disappeared from the Golgi region in cells treated with 1 μM M-COPA for 8 h (Fig. 1, *B* and *C*). Golgi matrix protein 130 kDa (GM130) dispersed to the cytosol without any change in its protein levels (Fig. S1, *B* and *C*), indicating that M-COPA affects ER–Golgi trafficking. M-COPA markedly increased the colocalization of KIT/PDGFRA with an ER marker, protein disulfide isomerase (PDI), suggesting that ER export of KIT and PDGFRA is blocked in M-COPA-treated GIST-T1 cells (Fig. 1, *B* and *C*). In PC-9 cells, EGFR and MET were found at the PM, the perinuclear area, and endosomal puncta (Fig. 1, *D* and *E*), as previously reported (30, 31, 32, 33). Like KIT/PDGFRA in GIST-T1 cells, EGFR and MET were retained in the ER of PC-9 cells in the presence of M-COPA (Fig. 1, *D* and *E*). Cell surface biotinylation and streptavidin pulldown assay, which measures PM levels of RTKs as immunoblot bands (34), showed the presence of RTKs in the PM (Fig. 1*F*). M-COPA treatment reduced the PM levels of RTKs to less than one-third, confirming that M-COPA blocks the biosynthetic secretory trafficking.

**Figure 2:**
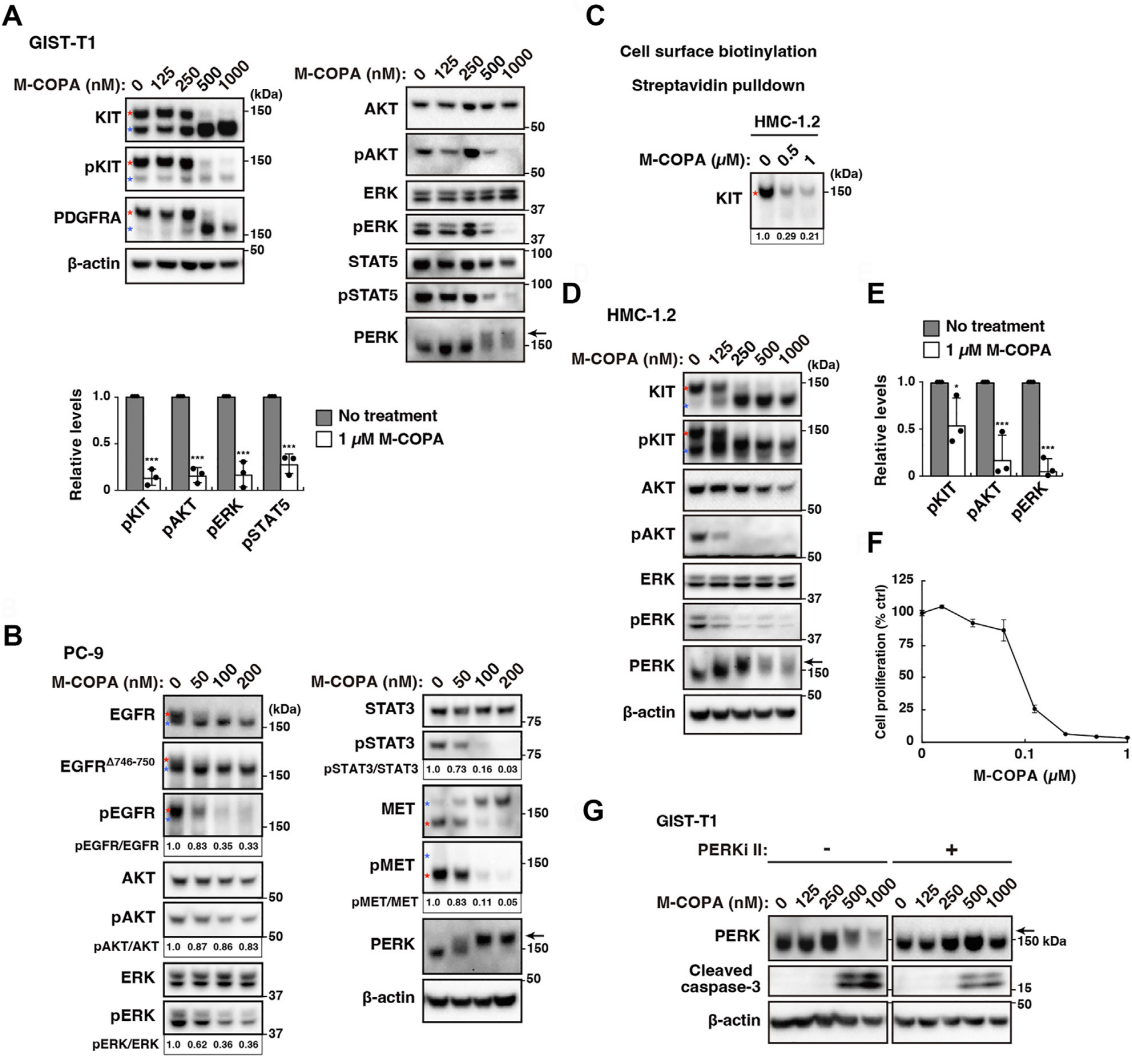
**M-COPA inhibits RTK activation by blocking receptor trafficking from the ER.***A* and *B*, GIST-T1 (*A*) and PC-9 (*B*) cells were treated M-COPA for 8 h and then immunoblotted. Relative band intensities normalized with each control sample are shown. *Red* and *blue asterisks* indicate the mature and immature RTKs, respectively. *Arrows* indicate phosphorylated PERK. The graph shows the relative levels of phospho-KIT (pKIT), pAKT, pERK, and pSTAT5. Results (%) represent means ± SD from three independent experiments. ∗∗∗*p* < 0.001, Student’s *t* test. Molecular size markers in kDa are indicated on the *right*. *C*–*E*, HMC-1.2 cells were treated with M-COPA for 8 h. *C*, cell surface KIT was treated with biotin. The biotinylated KIT was pulled down with streptavidin and subsequently immunoblotted. *D*, lysates were immunoblotted. *E*, the graph shows the relative levels of pKIT, pAKT, and pERK. Results (%) represent means ± SD from three independent experiments. ∗*p* < 0.05, ∗∗∗*p* < 0.001, Student’s *t* test. *F*, HMC-1.2 cells were treated with M-COPA for 48 h. Cell proliferation was assessed by ATP production. Values represent mean ± SD (*n* = 3). *G*, GIST-T1 cells were treated with M-COPA and/or 100 nM PERK inhibitor II (PERKi II) for 24 h and then immunoblotted for PERK, cleaved caspase-3, and β-actin. An *arrow* indicates phosphorylated PERK.

Immunoblotting showed that these four RTKs were found as doublet bands (Fig. 2, *A* and *B*); the upper bands of KIT, PDGFRA, and EGFR are mature forms, which appear after complex-glycosylation in the Golgi (red asterisks), and MET lower band is the mature form, which is proteolytically cleaved in the TGN (10, 35, 36). In M-COPA-treated cells, these RTKs were retained in immature forms unable to be transported from the ER (Fig. 2, *A* and *B*, blue asterisks). This trafficking inhibition was correlated with RTK dephosphorylation. In GIST-T1 cells, M-COPA treatment significantly decreased the levels of phospho-AKT (pAKT), pERK, and pSTAT5 (to 0.16 ± 0.07, 0.17 ± 0.12, and 0.28 ± 0.09, respectively), indicating that KIT could not cause downstream activation in the ER. Although the effect of M-COPA in PC-9 cells was generally similar to that in GIST-T1 cells, pAKT decreased by only 17% after M-COPA treatment (Fig. 2*B*). A previous study has reported that PC-9 cells lose the expression of phosphatase and tensin homolog (PTEN), which is a negative regulator of the PI3K-AKT pathway (28). This may explain the partial effects of M-COPA on pAKT in PC-9 cells observed in our study. The KIT mutant-expressing MCL cell line (37) HMC-1.2 also gave similar results as GIST-T1 (Fig. 2, *C*–*F*). In addition to cancer cells, a human skin fibroblast cell line Hs 925.Sk also contained immature RTKs in the presence of M-COPA (Fig. S2*A*). Interestingly, pAKT and pERK in the normal cells were unaffected. Indeed, compared with that of cancer cells, the growth sensitivity of Hs 925.Sk cells to M-COPA was quite low (Fig. S2*B*), indicating that Hs 925.Sk cells can activate growth signaling without PM-localized RTKs. Taken together, these results suggest that M-COPA inhibits the ER export of not only KIT but also PDGFRA, EGFR, and MET.

An immaturely membrane-bound glycosylated TGN protein 46-kDa (TGN46) was also increased in M-COPA-treated cells (Fig. S2*C*), suggesting that M-COPA blocks the general protein export from the ER.

### M-COPA can induce apoptosis in a PERK-independent manner in RTK-mutated cells

The blockade of ER–Golgi trafficking induces ER stress responses, such as PKR-like ER kinase (PERK) activation (26). In the presence of M-COPA, PERK was converted to its higher molecular weight form because of multiple phosphorylation (Figs. 2, *A*, *B*, and *D*, and S3*A*), indicating that M-COPA affects the secretory pathway of proteins from the ER.

Caspase-3, a key factor influencing cell death (38), was cleaved in GIST-T1 and HMC-1.2 cells treated with M-COPA for 24 h and 8 h, respectively, which is a sign of apoptosis (Figs. 2*G* and S3*A*). Previous studies have demonstrated that ER stress-induced PERK activation causes apoptosis of host cells (26). Thus, we investigated whether the induction of PERK activation is necessary for M-COPA-induced apoptosis using PERK inhibitor II (PERKi II), a compound that suppresses the kinase activity of PERK through competition with ATP (39). Figures 2*G* and S3*A* show that PERKi II completely suppressed the M-COPA-induced mobility shift of PERK bands, confirming that it blocks PERK activation. Interestingly, under these conditions, caspase-3 was also cleaved, suggesting that growth signaling inhibition through the blockade of ER export of mutant KIT is sufficient for M-COPA-induced apoptosis of GIST-T1 and HMC-1.2 cells. Immunoblotting results revealed that the protein levels of AKT and STAT5 were decreased (Fig. S3*B*), and fragments of these proteins were present in the lower molecular weight region. We suspected that these proteins were cleaved by caspases. When cells were treated with M-COPA plus a pan-caspase inhibitor, Z-VAD-FMK, which blocks the protease activity of caspases (40), AKT and STAT5 cleavage were suppressed (Fig. S3*B*). The effect of Z-VAD-FMK was confirmed by the reduction of completely cleaved caspase-3. Z-VAD-FMK addition could not restore the protein ERK levels decreased by M-COPA, indicating that M-COPA induces the activation of Z-VAD-FMK-sensitive proteases that cleave AKT and STAT5 but not ERK. Taken together, these results suggest that caspase-3 activation by M-COPA is independent of PERK activation but dependent on the blockade of ER export of mutant RTKs in cancer cells.

### Simultaneous inhibition of ARF1, ARF4, and ARF5 is required for mimicking the action of BFA/M-COPA

**Figure 3:**
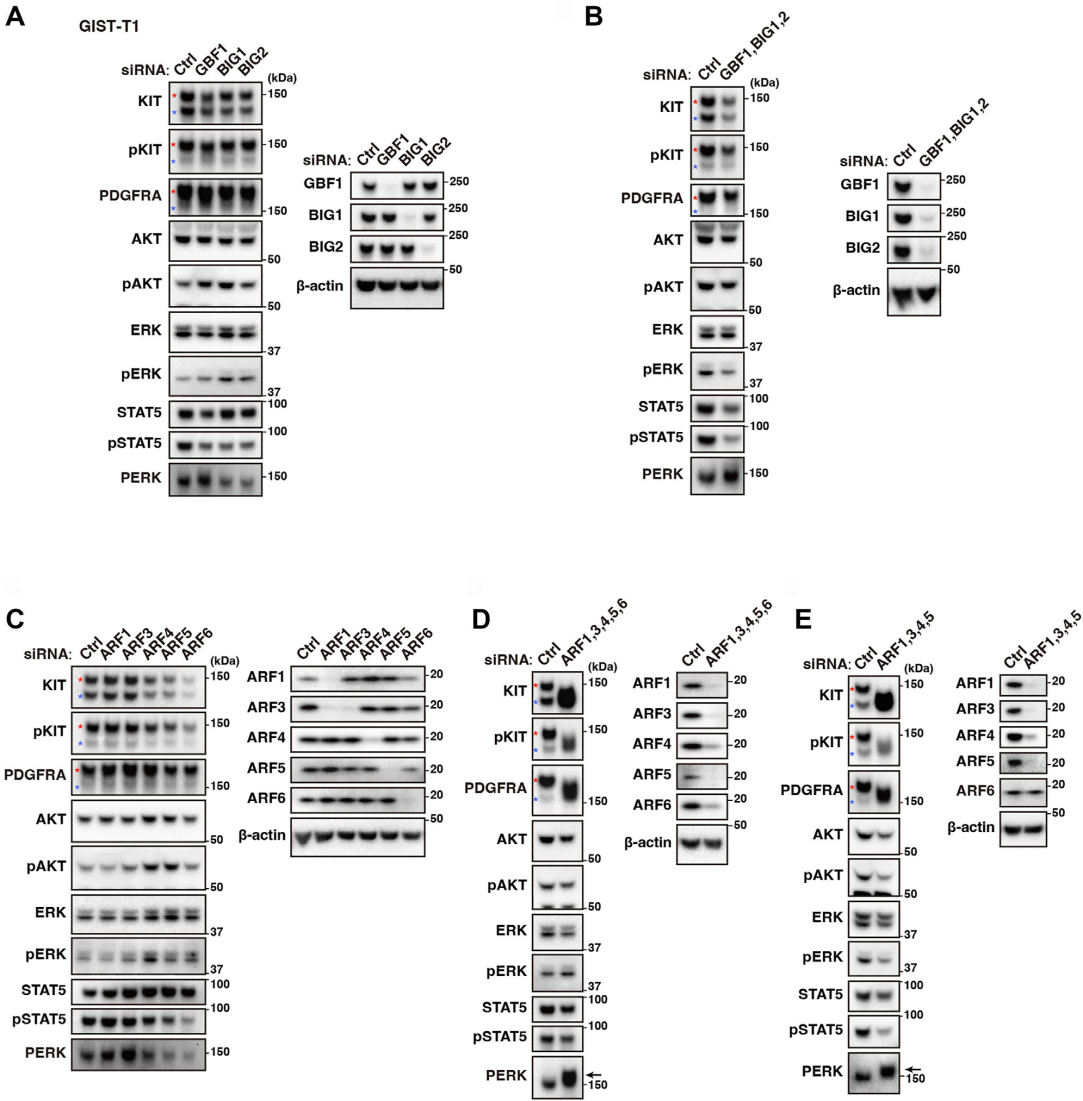
**Simultaneous knockdown of ARFs is required for mimicking BFA/M-COPA treatment in GIST-T1 cells.***A*–*E*, GIST-T1 cells were transfected with the indicated siRNA for 48 h. Lysates were immunoblotted with the indicated antibodies. *Red* and *blue asterisks* indicate the mature and immature RTKs, respectively. *Arrows* indicate phosphorylated PERK. Molecular size markers in kDa are indicated on the *right*.

Several studies have reported that BFA, golgicide A, and M-COPA block intracellular trafficking by suppressing the dissociation of ARF from GEF (18, 19, 20, 41, 42). Therefore, we explored whether knockdown of GEF and ARF with small interfering RNAs (siRNAs) can phenocopy BFA/M-COPA treatment to understand the precise targets of these inhibitors. Golgi-specific BFA-resistance guanine nucleotide exchange factor 1 (GBF1), BFA-inhibited guanine nucleotide-exchange protein 1 (BIG1), and BIG2 are widely known as BFA-sensitive GEFs (43). However, knockdown of GBF1, BIG1, or BIG2 did not exhibit similar results as shown by BFA/M-COPA treatment in the mobility shift of KIT/PDGFRA bands, signal inhibition, or PERK activation (Fig. 3*A*). Although simultaneous knockdown of GBF1, BIG1, and BIG2 slightly decreased the levels of KIT, pERK, and pSTAT5, their knockdown did not yield similar effects to that of BFA/M-COPA treatment (Fig. 3*B*), suggesting that the action of BFA/M-COPA cannot be explained by GBF1/BIG1/BIG2 inhibition alone. In other words, in addition to GBF1/BIG1/BIG2, other GEFs play a role in RTK trafficking from the ER. At present, the identification of BFA/M-COPA-sensitive GEFs is under way.

Next, we knocked down major ARF members, such as ARF1, ARF3, ARF4, ARF5, and ARF6. Like the results observed after GEF knockdown, single knockdown of an ARF member did not phenocopy M-COPA treatment (Fig. 3*C*). Although knockdown of ARF4, ARF5, and ARF6 decreased the levels of KIT and pSTAT5, the effect was not due to inhibition of ER export, as the accumulation of immature RTKs was not observed. Notably, ARF1 siRNA greatly reduced the ARF3 protein levels. As shown in Fig. S4*A*, M-COPA decreased the ARF3 level, indicating that ARF1 may play a role in the stability of ARF3 protein. Next, we simultaneously knocked down ARF1, ARF3, ARF4, ARF5, and ARF6. Interestingly, simultaneous knockdown of these ARFs showed the band shift of KIT, PDGFRA, and PERK activation, indicating that these RTKs are retained in the ER by ARF1–6 knockdown (Fig. 3*D*). ARF6 siRNA was not required for the blockade of ER export of the RTKs (Fig. 3*E*), suggesting that ARF6 is not involved in ER–Golgi trafficking.

**Figure 4:**
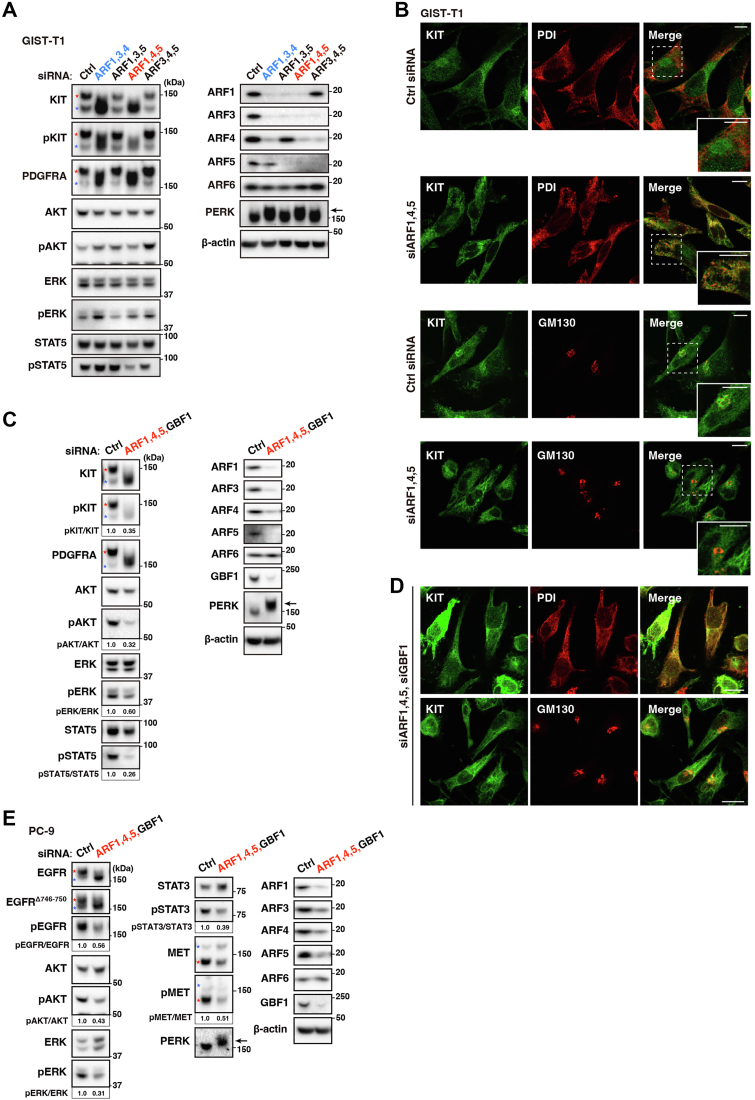
**At least simultaneous knockdown of ARF1, ARF4, and ARF5 is necessary for mimicking BFA/M-COPA treatment.***A*–*D*, GIST-T1 cells were transfected with the indicated siRNAs and then cultured for 48 h. *A* and *C*, lysates were immunoblotted with the indicated antibodies. Relative band intensities normalized with each control sample are shown. *Red* and *blue asterisks* indicate the mature and immature RTKs, respectively. *Arrows* indicate phosphorylated PERK. Molecular size markers in kDa are indicated on the *right*. *B* and *D*, cells were immunostained for KIT, PDI (ER marker), and Golgi matrix protein 130 kDa (GM130). Magnified images of the *boxed area* are shown. Scale bars indicate 10 μm and 20 μm in (*B*) and (*D*), respectively. Note that the effects of transfection of ARF1, ARF4, ARF5, and GBF1 siRNAs closely resembled the effects of BFA/M-COPA treatment. *E*, PC-9 cells were transfected with the indicated siRNAs for 24 h and then immunoblotted. An *arrow* indicates phosphorylated PERK.

We further determined the siRNA required for mimicking BFA/M-COPA treatment. As shown in Figure 4*A*, ER export of RTKs was not inhibited in the absence of ARF1 or ARF4 siRNA. ARF5 siRNA addition to ARF1/ARF4 siRNAs expedited the effect of BFA/M-COPA treatment (Fig. 4, *A* and *B*). We could not conclude whether ARF3 knockdown was required for phenocopying BFA/M-COPA treatment, because ARF1 knockdown decreased ARF3 levels, as shown in Figure 3*C*. Unlike M-COPA treatment, the simultaneous knockdown did not affect pAKT or pERK. A previous study showed that ARF inhibition with a dominant negative ARF mutant induces feed-forward stimulation of GBF1 (44). We expected that upon ARF1/4/5 knockdown, GBF1 would be recruited to the membrane, causing ARFs other than ARF1/4/5 to be activated as compensation. Therefore, in addition to ARF1/4/5 siRNAs, we transfected GBF1 siRNA to mimic BFA/M-COPA treatment. As shown in Figure 4, *C* and *D*, most effects of ARF1/4/5 siRNAs plus GBF1 siRNA were similar to that of BFA/M-COPA treatment: KIT and PDGFRA were retained in their immature forms in the ER, KIT signaling was inhibited (pKIT to 35%, pAKT to 32%, pERK to 60%, and pSTAT5 to 26%), and PERK was activated. However, unlike M-COPA treatment, the knockdown did not induce dispersion of GM130 (Fig. 4*D*, lower panels; compare with Fig. S1*B*), indicating that additional knockdown of ARFs/GEFs may be required for completely mimicking the BFA/M-COPA treatment. As shown in Figure 4*E*, ARF1/4/5 and GBF1 knockdown in PC-9 cells demonstrated similar results in that ER export of RTKs was blocked, resulting in the inactivation of EGFR. The simultaneous knockdown increased immature TGN46 protein (Fig. S4*B*), suggesting that ARF1/4/5 and GBF1 play an essential role not only in the trafficking of RTKs but also in that of other membrane-bound proteins. We also performed knockdown experiments with Dicer-substrate short interfering RNAs (DsiRNAs) (45), which have different sequences from the siRNAs shown in Figures 3 and 4. Single knockdown of ARF1, ARF4, ARF5, or GBF1 with DsiRNA did not cause mobility shift of RTK bands and PERK activation (Fig. S4, *C* and *D*). Similar to ARF1 siRNA, ARF1 DsiRNA decreased ARF3 protein level. Simultaneous transfection of ARF1/4/5 and GBF1 DsiRNAs blocked ER export of KIT and PDGFRA; caused KIT dephosphorylation; decreased pKIT (to 41%), pAKT (to 57%), pERK (to 33%), and pSTAT5 (to 64%) levels; and led to phosphorylation of PERK (Fig. S4, *E* and *F*). Taken together, these results suggest that the simultaneous inhibition of at least ARF1, ARF4, ARF5, and GBF1 is required for mimicking the effect of BFA/M-COPA treatment; in other words, RTKs require the simultaneous activation of ARF1, ARF4, ARF5, and GBF1 for their ER export.

### M-COPA inhibits activation and membrane localization of ARF1, ARF4, and ARF5

**Figure 5:**
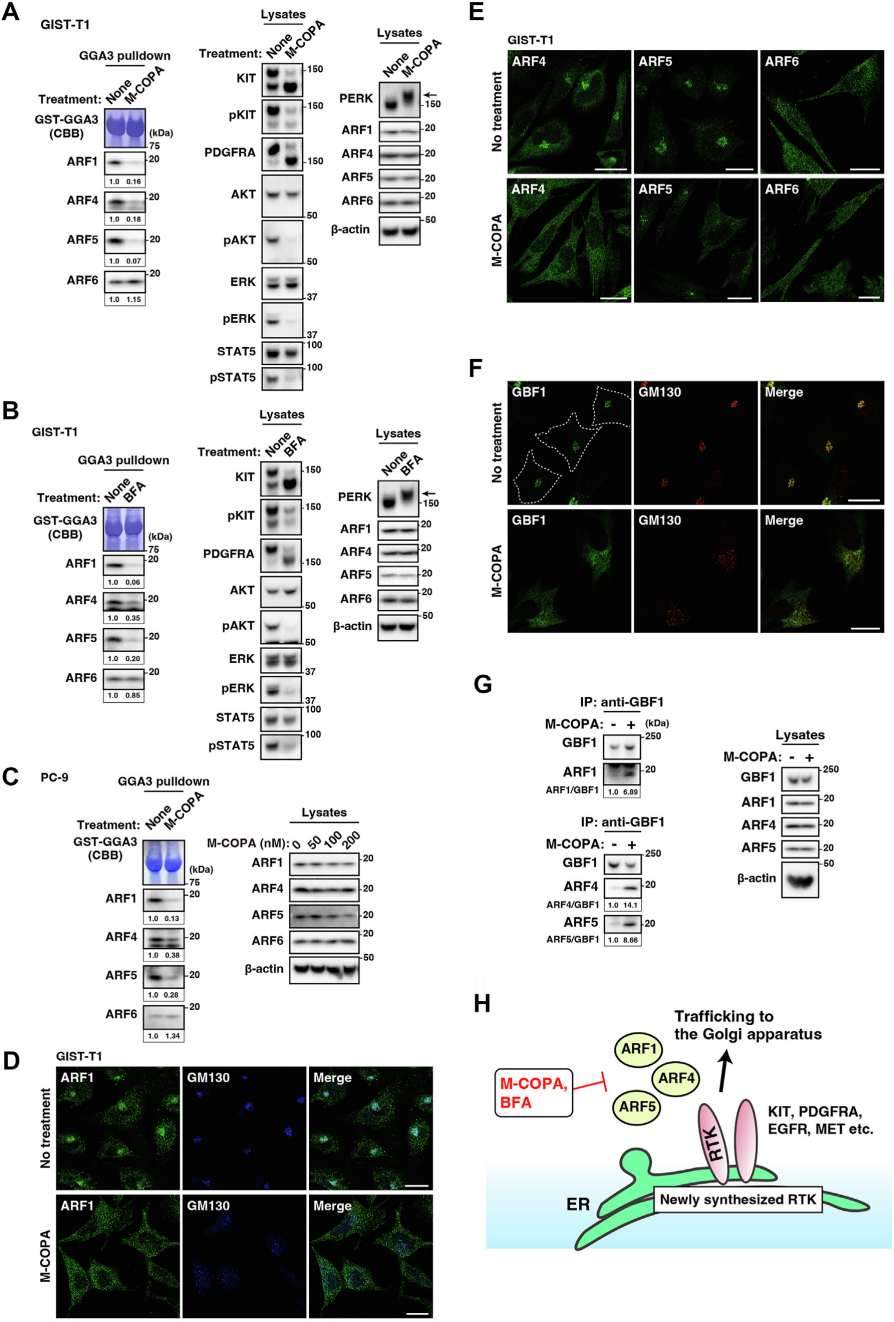
M**-COPA and BFA inhibit the activation of ARF1, ARF4, and ARF5 but not ARF6.***A* and *B*, GIST-T1 cells were treated with (*A*) 1 μM M-COPA or (*B*) 1 μM BFA for 4 h. ARFs were pulled down with GST-tagged GGA3(1–316) (GST-GGA3) and then immunoblotted (*left*). Amounts of GST-GGA3 were confirmed by CBB staining. Lysate immunoblots are shown in the *center* and *right side*. Relative band intensities normalized with each control sample are shown. *Arrows* indicate phosphorylated PERK. Molecular size markers in kDa are indicated on the *right*. *C*, PC-9 cells were treated with 200 nM M-COPA for 8 h. ARFs were pulled down with GST-GGA3 and then immunoblotted. Lysate immunoblots under the same condition are shown in the *right side* and Figure 2*B*. *D*–*F*, GIST-T1 cells treated with 1 μM M-COPA for 4 h were immunostained with the indicated antibody. *Dashed lines* indicate cell borders. Scale bars, 20 μm. *G*, GIST-T1 cells were treated with 1 μM M-COPA for 4 h. GBF1 was immunoprecipitated and then immunoblotted. Amounts of co-immunoprecipitated ARF are expressed relative to the control sample, after normalization with GBF1 levels. *H*, a schematic model of the ARF-dependent trafficking of RTKs from the ER. BFA and M-COPA inhibited the dissociation of ARF1, ARF4, and ARF5 from GBF1, resulting in the blockade of ER export of RTKs, such as KIT, PDGFRA, EGFR, and MET. Therefore, RTKs are exported from the ER in an ARF1/4/5-dependent manner. In addition, RTK mutants could not become fully active; thus, BFA and M-COPA inhibit the growth signaling in RTK-addicted cancer cells. GM130, Golgi marker; IP, immunoprecipitation.

Next, we investigated whether M-COPA inhibits ARF1/4/5. We performed an *in vitro* pulldown assay with glutathione S-transferase-fused Golgi-localized γ-adaptin ear-containing, ARF-binding protein 3 (GST-GGA3), which binds to active ARF (46, 47). As shown in Figure 5*A*, ARF1, ARF4, ARF5, and ARF6 were activated in GIST-T1 cells, although ARF3 pulldown was not detected probably because of a characteristic of ARF3 antibody. M-COPA reduced the levels of GGA3-bound ARF1, ARF4, and ARF5 to less than 18%. ARF6 binding was not blocked, suggesting that M-COPA specifically inhibits the activation of ARF1, ARF4, and ARF5. These pulldown assay data support the results of the knockdown experiment, indicating that ARF6 is not involved in ER export of RTKs. BFA treatment gave similar results to those of M-COPA treatment (Fig. 5*B*), suggesting that both these compounds can inhibit ARF1, ARF4, and ARF5. Furthermore, M-COPA inhibited ARF1, ARF4, and ARF5, but not ARF6, in PC-9 cells (Fig. 5*C*).

Next, we confirmed the localization of ARFs in M-COPA-treated cells. Previous studies have reported that active ARFs are recruited to organelles, whereas inactive ARFs are dissociated from the membranes to the cytosol (16, 48, 49). Under normal conditions, ARF1, ARF4, and ARF5 were mainly found in the perinuclear region (Figs. 5, *D* and *E* and S5). However, they relocated to the cytosol after M-COPA treatment, but ARF6 distribution was not affected by the treatment. These results supported the data of the GST pulldown assay. Next, we verified whether GBF1 activity was decreased in M-COPA-treated cells. Similar to ARF1/4/5, perinuclear GBF1 disappeared after M-COPA treatment (Fig. 5*F*). Because BFA immobilizes the ARF-GBF1 complexes (20, 41, 42), we performed a co-immunoprecipitation assay to examine whether M-COPA affected the association of GBF1 with ARFs. As shown in Figure 5*G*, co-immunoprecipitation of ARF1/ARF4/ARF5 with GBF1 was greatly increased by M-COPA treatment (ARF1, 6.89-fold; ARF4, 14.1-fold; and ARF5, 8.66-fold), indicating that GBF1 was indeed inhibited by the treatment. Taken together, these results suggest that BFA/M-COPA simultaneously inhibits ARF1, ARF4, and ARF5 by immobilizing their association with GBF1.

## Discussion

In this study, we demonstrated that simultaneous inhibition of at least ARF1, ARF4, and ARF5 is required for mimicking BFA/M-COPA treatment. In the knockdown condition in GIST cells, KIT mutant and PDGFRA are retained in the ER, where they are unable to reach their fully active form. EGFR mutant and MET in LAD cells are exported from the ER in a similar manner to KIT. These results suggest that these RTKs are exported from the ER towards the Golgi in a ARF1/4/5-dependent manner (Fig. 5*H*). Because the knockdown of only one ARF member does not mimic the effect of BFA/M-COPA, simultaneous inhibition of ARF1, ARF4, and ARF5 is essential for investigating the mechanism of action of these drugs.

Recent studies have reported that RTKs other than KIT, such as FLT3-ITD, PDGFRA, MET, and insulin-like growth factor receptor 1 (IGF-1R), also use the Golgi/TGN as their signaling platform in cancer cells (13, 33, 50, 51, 52). Moreover, non-RTK proto-oncogene products, such as Src-family kinases, RAS, and mTOR, can initiate growth signaling from the Golgi/TGN (10, 53, 54, 55, 56, 57). Therefore, these studies raise a possibility that inhibitors of protein trafficking from the ER to the Golgi can be potential candidates for anti-cancer drugs. Although the bioavailability of M-COPA is higher than that of BFA, the stability of M-COPA *in vivo* is not sufficient for a clinical trial. Thus, the development of M-COPA derivatives with considerably improved *in vivo* stability is now under way.

BFA and M-COPA cause PERK activation in GIST-T1 and HMC-1.2 cells, but the activation is not necessary for inducing apoptosis. In these cells, mutant KIT-dependent AKT activation has an essential role in the anti-apoptotic status (14, 58); thus, AKT inhibition through blockade of ER export of KIT is sufficient for inducing apoptosis. In addition to PERK, ATF6, and IRE1 pathways are also activated by ER stress (26). However, these ER stress-induced activations may not be essential for RTK-addicted cancer cells.

M-COPA treatment for a prolonged period may induce caspase-dependent limited proteolysis of AKT and STAT5 in GIST-T1 cells. The cleavage causes these proteins to lose survival signaling (59). Previous studies have shown that over 1000 proteins including AKT are cleaved by caspases (60, 61). In our study, cleaved STAT5 was present in M-COPA-treated cells, indicating that STAT5 is also a substrate candidate for caspases. Because AKT activation is essential for anti-apoptosis signaling in mutant KIT-addicted cancer cells (14, 58), the cleavage of AKT may serve as a positive feedback mechanism for M-COPA-induced apoptosis. Thus, once caspases are activated, intracellular AKT phosphorylation levels may be synergistically decreased in M-COPA-treated cells. Another possibility is that AKT and STAT5 are cleaved after apoptosis. Further studies are required to clarify whether degradation of AKT and STAT5 contributes to M-COPA-related growth suppression.

Our study showed that the growth sensitivity of normal cells to M-COPA is markedly lower than that of RTK-addicted cancer cells. M-COPA blocked ER export of EGFR, MET, and PDGFRA in Hs 925.Sk cells but could not inhibit AKT and ERK activation. We previously observed ligand-dependent KIT signaling in a normal mast cell line treated with M-COPA (15). Considering that M-COPA derivatives are being developed as an anti-cancer drug, the difference of sensitivity to M-COPA between cancer cells and normal cells is useful to avoid side effects.

Molecularly targeted drugs, such as a tyrosine kinase inhibitor (TKI) imatinib, can extend the life of patients with progressive GIST (62, 63). However, patients can develop resistance to imatinib within 2 to 3 years after treatment. Secondary mutations in the *KIT* gene, which causes KIT to lose sensitivity to TKIs, are frequently found in imatinib-resistant GISTs. Similarly, TKI resistance has also been found in EGFR-mutated LAD (3, 64). In this study, M-COPA inhibited KIT signaling of HMC-1.2 cells endogenously expressing KIT^D816V^, an imatinib-resistant mutant. Therefore, trafficking inhibition from the ER would be a promising strategy for the suppression of growth signaling through TKI-resistant RTK mutants.

Human ARF3 is known as a BFA-sensitive ARF member, which is structurally similar to ARF1; both are classified as class I ARFs (16, 43). However, we could not conclude in this study whether ARF3 knockdown is essential for mimicking BFA/M-COPA treatment because ARF1 depletion decreases the protein level of ARF3. Moreover, ARF3 antibodies used in this study are ineffective in GST-GGA3 pulldown assays and immunofluorescence. Further studies are required for elucidating the role of ARF3 in protein export from the ER.

Previous studies as well as this present study showed that BFA and M-COPA can disperse GM130 from the Golgi, indicating that these compounds affect the *cis*-side of the Golgi apparatus (42, 65). Although simultaneous knockdown of ARF1, ARF4, ARF5, and GBF1 blocks protein trafficking from the ER, the knockdown does not disperse GM130 from the Golgi area. A previous study reported that ARF4 but not ARF1/ARF5 plays a role in BFA-induced growth inhibition (66), indicating that ARF4 protein also influences the effects of BFA after treatment. This may be a reason why the simultaneous knockdown did not completely mimic BFA/M-COPA treatment. Furthermore, the simultaneous knockdown of GBF1, BIG1, and BIG2 also did not mimic BFA/M-COPA treatment. Knockdown of additional GEFs may be required to understand the mode of action of BFA/M-COPA. Thus, analyses of “additional hits” of GEFs and ARFs for fully understanding BFA/M-COPA action are currently in motion.

In conclusion, simultaneous inactivation of at least ARF1, ARF4, and ARF5 is required for mimicking BFA/M-COPA treatment. Our results provide evidence that M-COPA certainly inhibits ARF1, ARF4, and ARF5 but not ARF6. Moreover, from a clinical perspective, our study can contribute to the development of M-COPA and its derivatives as a molecular targeted drug that suppresses the growth of RTK-addicted cancers. Therefore, our study provides significant insights into intracellular trafficking in cancer cell biology and for molecular targeted therapeutics.

## Experimental procedures

### Cell culture

GIST-T1 (27) and Hs 925.Sk cells were procured from Cosmo Bio and American Type Culture Collection, respectively. The cells were cultured in Dulbecco’s Modified Eagle’s Medium (DMEM) supplemented with 10% fetal calf serum (FCS) and penicillin and streptomycin (Pen/Strep) at 37 °C. PC-9 (European Collection of Authenticated Cell Cultures) and HMC-1.2 cells (37) were cultured at 37 °C in RPMI1640 supplemented with 10% FCS and Pen/Strep. To culture HMC-1.2 cells, 50 μM 2-mercaptoethanol was added. All human cell lines were tested for *Mycoplasma* contamination using a MycoAlert *Mycoplasma* Detection Kit (Lonza).

### Cell proliferation assay

Cells were cultured with M-COPA for 48 h. Cell proliferation was quantified using the CellTiter-Glo Luminescent Cell Viability Assay (Promega), according to the manufacturer’s instructions. ATP production was measured with the 2030 ARVO X3 Multilabel Plate Reader (PerkinElmer) or Synergy H1 Multimode Microplate Reader (Agilent).

### Antibodies

The lists of antibodies used for immunoblotting, immunoprecipitation, and immunofluorescence are shown in Table S1.

### Chemicals

Z-VAD (Ome)-FMK (Abcam), PERKi II (Sigma-Aldrich), imatinib mesylate (Cayman Chemical), and M-COPA (22, 23) were dissolved in dimethyl sulfoxide. BFA (Sigma-Aldrich) was dissolved in ethanol.

### Gene silencing with siRNA

To silence the *ARF* and *GEF* genes, ON-TARGETplus SMARTpool siRNAs and DsiRNAs were purchased from Horizon Discovery and Integrated DNA Technologies (Coralville), respectively. A list of the siRNAs and DsiRNAs used in this study is provided in Table S1. Electroporation was performed using the NEON Transfection System (Thermo Fisher Scientific), according to the manufacturer’s instructions.

### Immunofluorescence confocal microscopy

GIST-T1 and PC-9 cells were cultured on poly L-lysine-coated coverslips separately and fixed with 4% paraformaldehyde for 20 min at room temperature. The fixed cells were permeabilized and blocked for 30 min in Dulbecco’s phosphate-buffered saline (D-PBS(−)) supplemented with 0.1% saponin and 3% bovine serum albumin (BSA) and then incubated with primary and secondary antibodies for 1 h each. After washing with D-PBS(−), the cells were mounted with Fluoromount (Diagnostic BioSystems). Confocal images were obtained with a FLUOVIEW FV10i (Olympus, Tokyo, Japan) or TCS SP5 II/SP8 (Leica) laser scanning microscope. Composite figures were prepared using FLUOVIEW FV1000 Viewer (Olympus), Leica Application Suite X Software (Leica), Photoshop, and Illustrator software (Adobe).

### Western blotting

Lysates prepared in sodium dodecyl-sulfate polyacrylamide gel electrophoresis (SDS-PAGE) sample buffer were subjected to SDS-PAGE and electrotransferred onto polyvinylidene fluoride membranes. Briefly, 5% skim milk in Tris-buffered saline with Tween 20 (TBST) was used to dilute the antibodies. For immunoblotting with anti-pKIT, anti-pEGFR, or anti-pMET, the antibodies were diluted with 3% BSA in TBST. Immunodetection was performed using Immobilon Western Chemiluminescent HRP Substrate (Sigma-Aldrich). Sequential re-probing of membranes was performed after complete removal of antibodies with Restore PLUS Western Blot Stripping Buffer (Thermo Fisher Scientific) or inactivation of peroxidase by 0.1% NaN_3_. The results were analyzed using ChemiDoc XRC+ with the Image Lab software (Bio-Rad). To immunoblot for cleaved caspase-3 of GIST-T1 cells, cells that were detached from the bottom of the dish and adherent cells were collected and then lysed. Total protein levels were confirmed by β-actin immunoblots and Coomassie brilliant blue staining.

### Immunoprecipitation

Lysates from 2 to 4 × 10^6^ cells were prepared in NP-40 lysis buffer supplemented with 50 mM HEPES (pH 7.4), 10% glycerol, 0.1 to 1% NP-40, 4 mM EDTA, 100 mM NaF, 1 mM Na_3_VO_4_, protease inhibitor cocktail, 2 mM β-glycerophosphate, 2 mM sodium pyrophosphate, and 1 mM phenylmethylsulfonyl fluoride. Immunoprecipitation was performed at 4 °C for 5 h using Dynabeads Protein G beads precoated with anti-GBF1 antibody. The immunoprecipitates were dissolved in the SDS-PAGE sample buffer and then immunoblotted.

### GST-GGA3(1–316) pulldown assay

Lysates from 2 to 4 × 10^6^ cells were prepared in lysis buffer supplemented with 10 mM NaF, 1 mM Na_3_VO_4_, protease inhibitor cocktail, 2 mM β-glycerophosphate, 2 mM sodium pyrophosphate, and 1 mM phenylmethylsulfonyl fluoride. According to the manufacturer’s instructions (Cytoskeleton), ARFs in cell lysates were incubated with GST-GGA3(1–316)-coated Sepharose beads at 4 °C for 1 h. These beads were washed once and then immersed in an SDS-PAGE sample buffer. Eluted proteins were resolved by SDS-PAGE and immunoblotted. Amounts of GST-GGA3(1–316) were checked by CBB staining.

### Cell surface biotinylation

M-COPA-treated cells were incubated with 0.2 mg/ml EZ-Link Sulfo-NHS-SS-Biotin (Thermo Fisher Scientific) for 45 min. After washing with D-PBS(−), the cells were incubated with 50 mM NH_4_Cl in D-PBS(−) for 10 min to quench biotin. Lysates were prepared in NP-40 lysis buffer. Subsequently, biotinylated proteins were pulled down with Streptavidin Agarose (Sigma-Aldrich) for 5 h, followed by elution with SDS-PAGE sample buffer. The above steps were performed at 4 °C to prevent intracellular trafficking.

### Statistical analysis

Differences between two groups were analyzed using a two-tailed Student’s *t* test. A value of *p* < 0.05 was considered statistically significant.

## Data availability

All data are contained in this article.

## Supporting information

This article contains supporting information.

## Conflict of interest

The authors declare that they have no conflicts of interest with the contents of this article.
