# Supplementary material for: Brefeldin A and M-COPA block the export of RTKs from the endoplasmic reticulum via simultaneous inactivation of ARF1, ARF4, and ARF5
Source: J Biol Chem. 2024 Apr 26;300(6):107327. doi: 10.1016/j.jbc.2024.107327 (PMC11127164; doi:10.1016/j.jbc.2024.107327)
Supplement: Supplemental Table [file mmc1.docx]

| Reagent  Supplementary Table 1 | Source | Catalog # |
| --- | --- | --- |
| Antibodies | | |
| AKT (clone 55) Mouse mAb | BD Transduction Laboratories | #610860 |
| AKT (pan) (40D4) Mouse mAb | Cell Signaling Technology | #2920 |
| Phospho-AKT (T308) (C31E5E) Rabbit mAb | Cell Signaling Technology | #2965 |
| ARF1 (ARFS 1A9/5) Mouse mAb | Santa Cruz Biotechnology | sc-53168 |
| ARF1 (1C10E3) Mouse mAb | Proteintech | #68069-1-Ig |
| ARF1 Rabbit polyclonal ab | Proteintech | #10790-1-AP |
| ARF3 (clone 41) Mouse mAb | BD Transduction Laboratories | #610784 |
| ARF3 (clone 41) Mouse mAb | Santa Cruz Biotechnology | sc-135841 |
| ARF4 Rabbit polyclonal ab | Proteintech | #11673-1-AP |
| ARF5 (14-07) Mouse mAb | Santa Cruz Biotechnology | sc-81893 |
| ARF5 Rabbit polyclonal ab | GeneTex | GTX104783 |
| ARF6 (3A-1) Mouse mAb | Santa Cruz Biotechnology | sc-7971 |
| ARF6 Rabbit polyclonal ab | Proteintech | #20225-1-AP |
| BIG1 (G-3) Mouse mAb | Santa Cruz Biotechnology | sc-376790 |
| BIG2 (H-6) Mouse mAb | Santa Cruz Biotechnology | sc-398042 |
| Cleaved Caspase-3 (Asp175) Rabbit polyclonal ab | Cell Signaling Technology | #9661 |
| EGFR (F4) Mouse mAb | Santa Cruz Biotechnology | sc-53274 |
| EGFR (D38B1) Rabbit mAb | Cell Signaling Technology | #4267 |
| EGFRΔ746-750 (D6B6) Rabbit mAb | Cell Signaling Technology | #2085 |
| EGFR [pY1068] Rabbit polyclonal ab | Cell Signaling Technology | #2234 |
| ERK1/2 (137F5) Rabbit mAb | Cell Signaling Technology | #4695 |
| ERK2 (K-23) Rabbit polyclonal ab | Santa Cruz Biotechnology | sc-153 |
| ERK [pT202/pY204] (E-10) Mouse mAb | Cell Signaling Technology | #9106 |
| ERK [pY204] (E-4) Mouse mAb | Santa Cruz Biotechnology | sc-7383 |
| GBF1 (clone 25) Mouse mAb | Santa Cruz Biotechnology | sc-136240 |
| GBF1 (clone 25) Mouse mAb | BD Transduction Laboratories | #612116 |
| GM130 (clone 35) Mouse mAb | BD Transduction Laboratories | #610823 |
| GM130 (EP892Y) Rabbit mAb | Abcam | ab52649 |
| Golgin-97 (CDF4) Mouse mAb | Thermo Fisher Scientific | #14-9767-82 |
| KIT (D13A2) Rabbit mAb | Cell Signaling Technology | #3074 |
| KIT (D3W6Y) Rabbit mAb | Cell Signaling Technology | #37805 |
| KIT (E-1) Mouse mAb | Santa Cruz Biotechnology | sc-17806 |
| KIT (clone 28) Mouse mAb | BD Transduction Laboratories | #612318 |
| Phospho-KIT (Y703) (D12E12) Rabbit mAb | Cell Signaling Technology | #3073 |
| MET (D1C2) Rabbit mAb | Cell Signaling Technology | #8198 |
| MET (D-4) Mouse mAb | Santa Cruz Biotechnology | sc-514148 |
| MET [pY1234/1235] (D26) Rabbit mAb | Cell Signaling Technology | #3077 |
| PDGFRA (D13C6) Rabbit mAb | Cell Signaling Technology | #5241 |
| PDGFRA (C-9) Mouse mAb | Santa Cruz Biotechnology | sc-398206 |
| PDI (RL90) Mouse mAb | Abcam | ab2792 |
| PERK (B-5) Mouse mAb | Santa Cruz Biotechnology | sc-377400 |
| STAT3 (124H6) Mouse mAb | Cell Signaling Technology | #9139 |
| Phospho-STAT3 (Tyr705) (D3A7) Rabbit mAb | Cell Signaling Technology | #9145 |
| STAT5 (D2O6Y) Rabbit mAb | Cell Signaling Technology | #94205 |
| STAT5 (clone 89) Mouse mAb | BD Transduction Laboratories | #610192 |
| Phospho-STAT5 (Tyr694) (D47E7) Rabbit mAb | Cell Signaling Technology | #4322 |
| TGN46 Rabbit polyclonal ab | Proteintech | #13573-1-AP |
| Donkey HRP anti-mouse IgG | Jackson ImmunoResearch | #715-035-151 |
| Donkey HRP anti-rabbit IgG | Jackson ImmunoResearch | #711-035-152 |
| Donkey anti-Mouse IgG (H+L), Alexa Fluor 488 | Thermo Fisher Scientific | A-21202 |
| Donkey anti-Rabbit IgG (H+L), Alexa Fluor 488 | Thermo Fisher Scientific | A-21206 |
| Donkey anti-Mouse IgG (H+L), Alexa Fluor 568 | Thermo Fisher Scientific | A-10037 |
| Donkey anti-Rabbit IgG (H+L), Alexa Fluor 568 | Thermo Fisher Scientific | A-10042 |
| Donkey anti-Mouse IgG (H+L), Alexa Fluor 647 | Thermo Fisher Scientific | A-31571 |
| Donkey anti-Rabbit IgG (H+L), Alexa Fluor 647 | Thermo Fisher Scientific | A-31573 |
| Oligonucleotides | | |
| ON-TARGETplus Human ARF1 (375) siRNA - SMARTpool | Horizon Discovery | L-011580-00 |
| ON-TARGETplus Human ARF3 (377) siRNA - SMARTpool | Horizon Discovery | L-011581-00 |
| ON-TARGETplus Human ARF4 (378) siRNA - SMARTpool | Horizon Discovery | L-011582-00 |
| ON-TARGETplus Human ARF5 (381) siRNA - SMARTpool | Horizon Discovery | L-011584-00 |
| ON-TARGETplus Human ARF6 (382) siRNA – SMARTpool | Horizon Discovery | L-004008-00 |
| ON-TARGETplus Human BIG1 (10565) siRNA – SMARTpool | Horizon Discovery | L-012207-00 |
| ON-TARGETplus Human BIG2 (10564) siRNA – SMARTpool | Horizon Discovery | L-012208-02 |
| ON-TARGETplus Human GBF1 (8729) siRNA – SMARTpool | Horizon Discovery | L-019783-00 |
| Negative control siRNA | Invitrogen | #4390843 |
| DsiRNA ARF1 | Integrated DNA Technologies | #109011723 |
| DsiRNA ARF4 | Integrated DNA Technologies | #109011731 |
| DsiRNA ARF5 | Integrated DNA Technologies | #109011751 |
| DsiRNA GBF1 | Integrated DNA Technologies | #109011759 |
| Negative Control DsiRNA | Integrated DNA Technologies | #51-01-14-03 |
