# Supplementary material for: Brefeldin A and M-COPA block the export of RTKs from the endoplasmic reticulum via simultaneous inactivation of ARF1, ARF4, and ARF5
Source: J Biol Chem. 2024 Apr 26;300(6):107327. doi: 10.1016/j.jbc.2024.107327 (PMC11127164; doi:10.1016/j.jbc.2024.107327)
Supplement: Supplemental Figures [file mmc2.pdf]

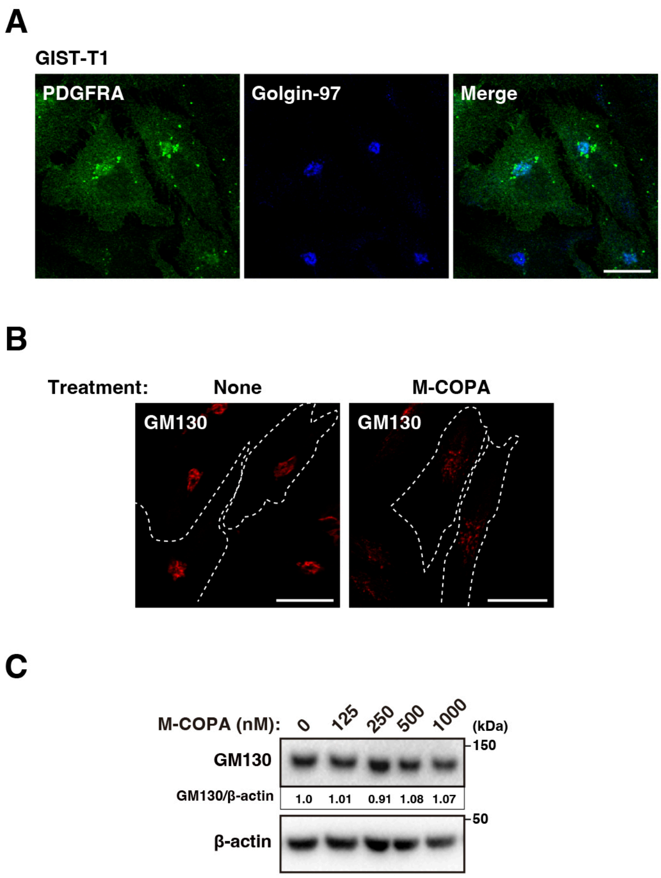

Figure S1. M-COPA affects the distribution of GM130.

A, GIST-T1 cells were immunostained for PDGFRA and golgin-97. Scale bar, 20 µm.

B and *C,* GIST-T1 cells were treated with 1 µM M-COPA for 8 h. *B,* Cells were immunostained with Golgi matrix protein 130 kDa (GM130). Dashed lines indicate cell borders. Scale bars, 20 µm. *C,* Lysates were immunoblotted for GM130 and β-actin. Amounts of GM130 are expressed relative to the control sample, after normalization with β-actin levels .Molecular size markers in kDa are indicated on the right.

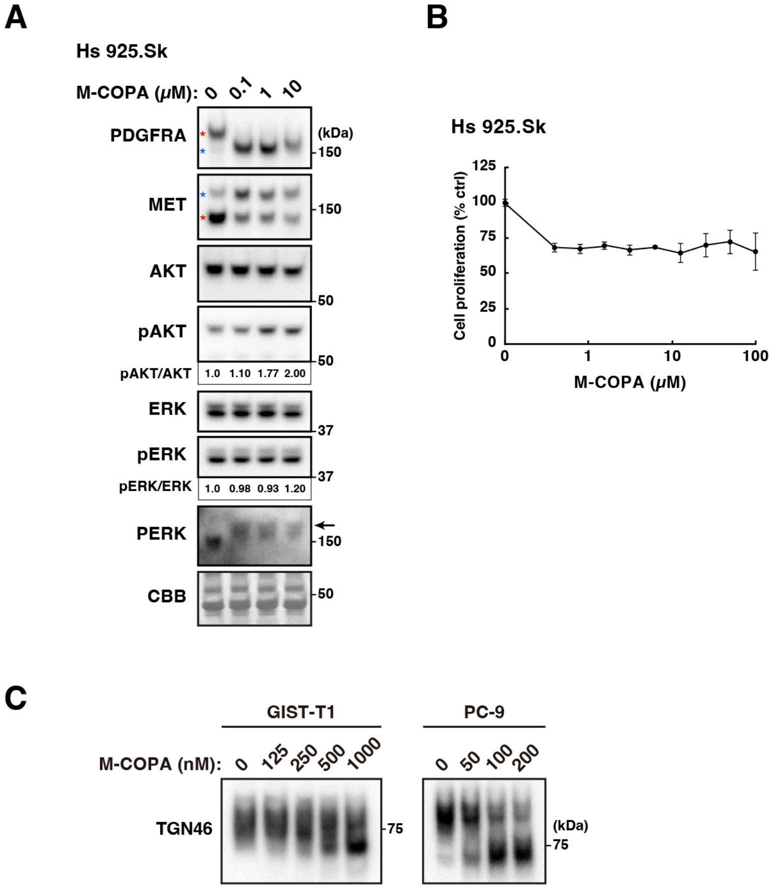

Figure S2. M-COPA blocks the protein trafficking from the ER to the Golgi apparatus.

A, Hs925. Sk cells were treated with M-COPA for 8 h then immunoblotted. Relative band intensities normalized with each control sample are shown. Molecular size markers in kDa are indicated on the right.

B, Hs925. Sk cells were treated with M-COPA for 48 h. Cell proliferation was assessed by ATP production. Values represent mean ± SD (n = 3).

C, GIST-T1 (left) and PC-9 cells (right) were treated with M-COPA for 8 h then immunoblotted for *trans*-Golgi network protein 46-kDa (TGN46).

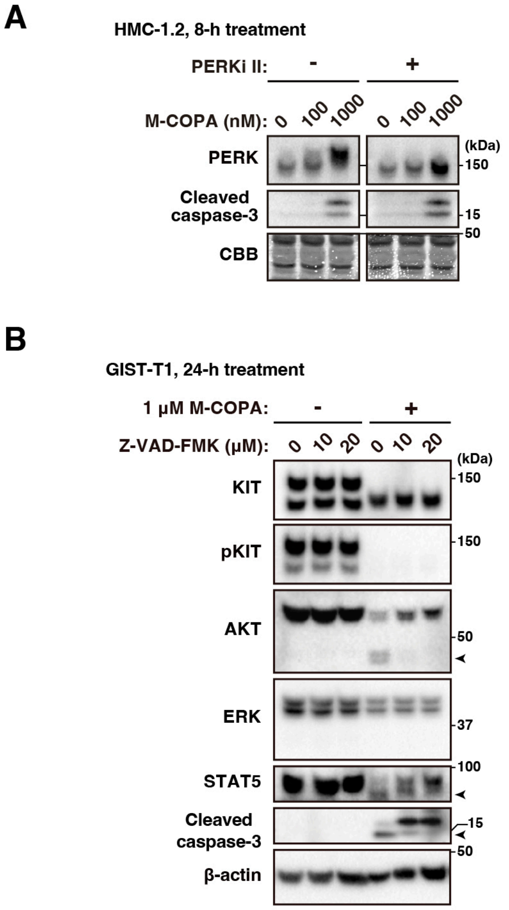

Figure S3. Activation of PERK is not required for M-COPA-induced apoptosis in HMC-1.2 cells.
A, HMC-1.2 cells were treated with M-COPA and/or 1 *μ*M PERK inhibitor II (PERKi II) for 24 h and then immunoblotted for PERK and cleaved caspase-3. Total protein levels were confirmed by CBB staining. Molecular size markers in kDa are indicated on the right.
B, GIST-T1 cells were treated with M-COPA and/or Z-VAD-FMK (pan-caspase inhibitor) for 24 h and immunoblotted. Arrowheads indicate the cleaved form of AKT, STAT5, and caspase-3.

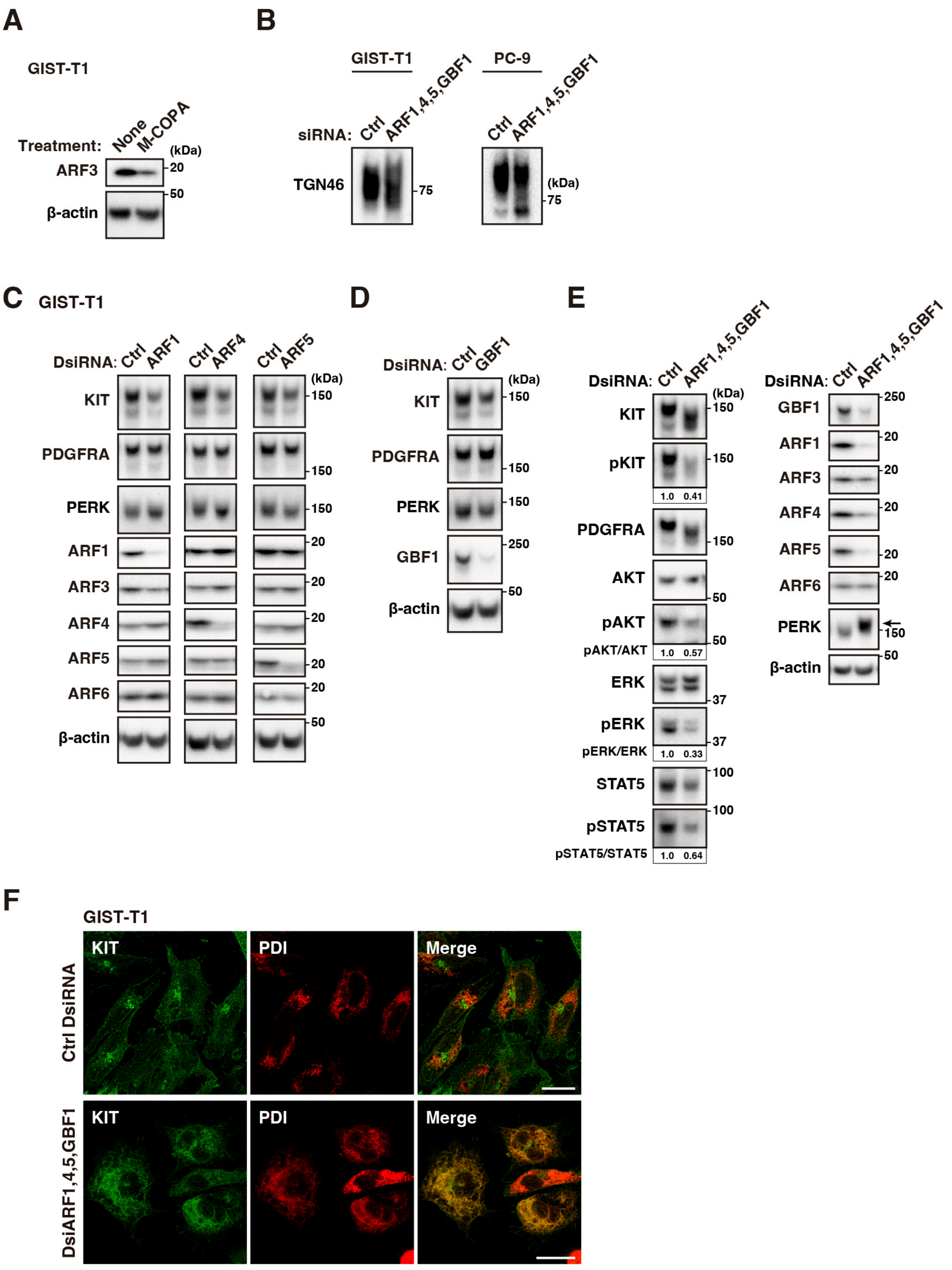

Figure S4. Simultaneous knockdown of ARF1, ARF4, ARF5, and GBF1 induces blockade of ER export of RTKs.

A, GIST-T1 cells were treated with 1 μM M-COPA for 24 h then immunoblotted for ARF3. Molecular size markers in kDa are indicated on the right.

B, GIST-T1 cells (left) or PC-9 cells (right) were transfected with the indicated siRNAs for 48 h then immunoblotted for *trans*-Golgi network protein 46-kDa (TGN46).

C-F, GIST-T1 cells were transfected with the indicated Dicer-Substrate Short Interfering RNAs (DsiRNAs) for 48 h. *C-E,* Lysates were immunoblotted. Relative band intensities normalized with each control sample are shown. An arrow indicates phosphorylated PERK. *F,* Cells were immunostained for KIT (green) and protein disulfide isomerase (PDI, ER marker, red). Scale bars, 20 μm.

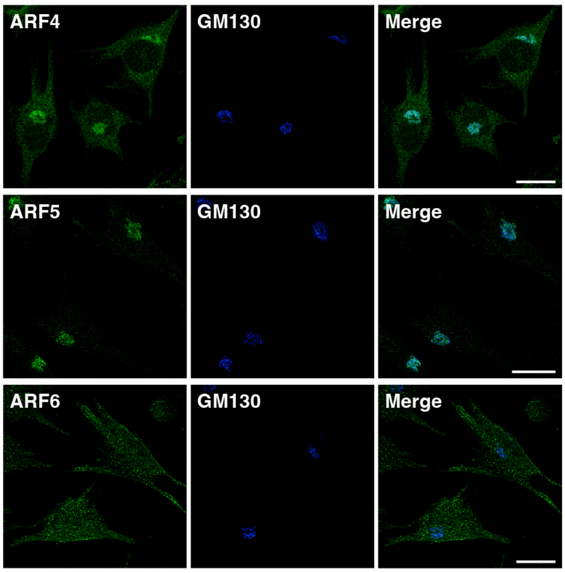

Figure S5. ARFs are localized in the perinuclear region and the plasma membrane.
GIST-T1 cells were immunostained for ARFs and Golgi matrix protein 130 kDa (GM130, Golgi marker, blue). Scale bars, 20 μm.
